# Raptor wing morphing with flight speed

**DOI:** 10.1098/rsif.2021.0349

**Published:** 2021-07-14

**Authors:** Jorn A. Cheney, Jonathan P. J. Stevenson, Nicholas E. Durston, Masateru Maeda, Jialei Song, David A. Megson-Smith, Shane P. Windsor, James R. Usherwood, Richard J. Bomphrey

**Affiliations:** ^1^ Structure and Motional Laboratory, Royal Veterinary College, Hatfield AL9 7TA, UK; ^2^ Department of Aerospace Engineering, University of Bristol, Bristol BS8 1TR, UK; ^3^ School of Mechanical Engineering, Dongguan University of Technology, Guangdong, People's Republic of China; ^4^ Interface Analysis Centre, School of Physics, University of Bristol, Bristol BS8 1TL, UK

**Keywords:** wing morphing, bird wings, three-dimensional reconstruction, bird aerodynamics

## Abstract

In gliding flight, birds morph their wings and tails to control their flight trajectory and speed. Using high-resolution videogrammetry, we reconstructed accurate and detailed three-dimensional geometries of gliding flights for three raptors (barn owl, *Tyto alba*; tawny owl, *Strix aluco*, and goshawk, *Accipiter gentilis*). Wing shapes were highly repeatable and shoulder actuation was a key component of reconfiguring the overall planform and controlling angle of attack. The three birds shared common spanwise patterns of wing twist, an inverse relationship between twist and peak camber, and held their wings depressed below their shoulder in an anhedral configuration. With increased speed, all three birds tended to reduce camber throughout the wing, and their wings bent in a saddle-shape pattern. A number of morphing features suggest that the coordinated movements of the wing and tail support efficient flight, and that the tail may act to modulate wing camber through indirect aeroelastic control.

## 1. Introduction

Birds fly over a large speed range morphing their wings and tail to modulate aerodynamic force production. At their fastest glide speeds, birds fold and sweep back their wings, and fully contract their tail [1]. At slower speed, first the wings unfold and sweep forward, spreading laterally, and at even slower speed, the tail spreads, in addition to the wings [2–4]. Avian wing morphing is more elaborate than unfolding and sweeping [5], but our detailed knowledge is largely limited to gross in-plane movements visible in the silhouette when viewed from above or below.

Despite limitations in our knowledge of avian wing morphing, each of the effects identified are broadly consistent with achieving efficient flight. Wing sweep alone, with minimal area change, allows wings to operate at high lift-to-drag ratios over a wide range of speeds [6]. When changes in wing sweep are combined with either wing area [7], tail area [8] or camber profile [9], the flight envelope can be enhanced further [9]. Additionally, tail morphing alone can also reduce the cost of flight through changing pitch or tail spread [10] because the tail plays an important role in modulating the lift distribution over the bird [11]. Related, when an avian-inspired robot, capable of wing sweep and tail contraction, was optimized for the power cost of flight, it used a broadly similar pattern of coordination with speed to that of birds [8].

The morphing described above can be broadly categorized as rigid-body rotations of a lifting surface, and changes in its shape. Wing sweep and tail pitch describe rotations, while tail spread, wing area or wing camber describe changes in shape. The latter can be further refined into changes within the plane of the lifting surface, such as area, or out of the plane, such as camber. As bird wings are highly coupled by passive mechanisms constraining the elbow, wrist and flight feathers to move in unison, shape change will generally be small and multi-dimensional, and result in a smooth aerofoil [12–15] as opposed to the discrete movable surfaces of most commercial aircraft, but note covert feathers can produce discontinuous surfaces in flying birds [16].

Here, we seek to understand how birds morph their wing and tail configuration over a range of self-selected gliding speeds, while also maintaining approximate weight support and moment balance. We use this conceptual framework for morphing to assess better the contributions of aeroelastic deflection of the feathers, planform shape change by the forelimb muscles, and articulation between the body and wings or tail; such parameters are critical for minimizing drag, changing the forces and moments produced by wings, and modifying stability [8,17–19].

**Figure 1.**
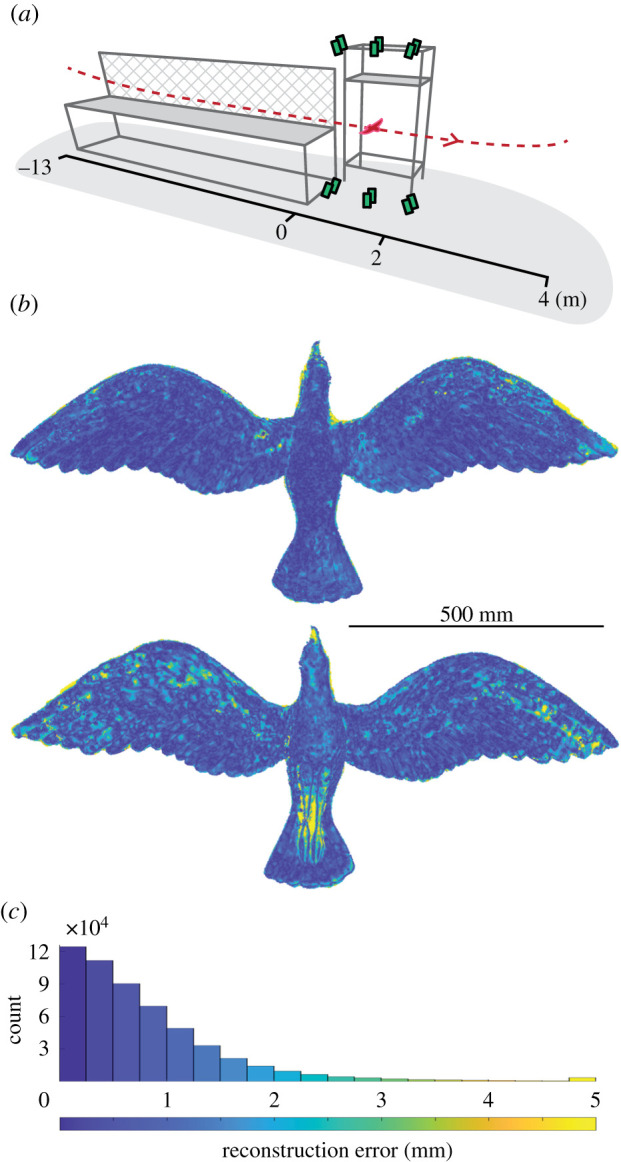
Bird geometry measurement apparatus and accuracy of its calibration. (*a*) The birds flew through an indoor flight corridor with their path (dashed red line) constrained by one mesh wall (hatched) and one wall of the building (not shown). Twelve high-speed cameras (green boxes) recorded images of the lower or upper surface of the gliding bird (outlined in red). (*b*) Reconstructions of a rigid model bird correspond well to its laser scan (top: dorsal surface; bottom: ventral surface). (*c*) Error in the reconstruction of the model bird is generally within a millimetre as seen in the error distribution. (*b*,*c*) Colour bar at bottom.

The gliding flights we examined cover a range of self-selected speeds in three species of raptor: a barn owl, *Tyto alba*, a tawny owl, *Strix aluco*, and a goshawk, *Accipiter gentilis*. The gliding flights occurred indoors in quiescent air. We describe only steady glides and within each glide, a single instance in time, for 13–15 glides for each bird. We measured wing shape and posture using photogrammetric methods (figure 1; similar to those in [5]), which provided detailed three-dimensional geometry of the entire bird. Our focus, here, is on the changes in wing posture and shape that we observe correlating with flight speed; however, we also provide a brief description of the similarities in the average shape and posture. A more detailed description of average wing shape and posture can be found in the electronic supplementary material.

## 2. Methods

### 2.1. Birds

The barn owl (*T. alba*), tawny owl (*S. aluco*) and goshawk (*A. gentilis*) were captive-bred and trained to fly between handlers on command and they received a food reward after each flight. The birds weighed 333 ± 7 g, 366 ± 2 g and 966 ± 2 g, respectively (mean ± standard error of the mean, s.e.m.).

### 2.2. Experimental overview

Flight measurements were conducted in a 17 m-long indoor flight corridor at the Royal Veterinary College (Hatfield, UK). The corridor was 2 m wide, bounded by a structural wall on one side and framed mesh on the other, with a suspended floor to elevate the flight path of the bird and permit camera views from above and below (figure 1*a*). In each flight, the birds flapped to build speed before entering a smooth glide, during which the measurements were taken. They subsequently entered a deceleration phase and finally perched 3–5 m beyond the measurement region. We only report on ‘steady’ glides where wing movements relative to the body could not be readily perceived in the reconstructions.

We imaged the birds using an array of 12, synchronized, high-speed cameras recording at 500 frames s^−1^, arranged in upper and lower sets (figure 1), comprising pairs of either Photron FASTCAM SA3 (1024 × 1024 pixels), FASTCAM SA-Z (1024 × 1024 pixels) or FASTCAM Mini WX100 (2048 × 2048 pixels) models (Photron Europe Limited, West Wycombe, UK). Camera placement ensured that all cameras viewed either the ventral or dorsal surface when the bird was located in the centre of the measurement volume. The horizontal cross-section through the measurement volume at gliding height was approximately 2 × 2 m. Custom stroboscopic LED lamps (comprising CoB LEDs of Bridgelux Vero 29; Digi-key Electronics, Thief River Falls, USA) illuminated the birds. Where possible, the imaging background was covered in black material to facilitate automated masking of the bird during later image processing. Birds were tagged with retroreflective motion-capture-marker stickers, visible in the reconstructions as white discs. Data from the motion-capture markers were not used in the analysis presented here.

### 2.3. Camera calibration

Camera calibration involved three steps: (i) intrinsic calibration; (ii) individual extrinsic calibration of upper and lower cameras sets and (iii) alignment of both sets' coordinate systems to the corridor reference frame. (i) The intrinsic properties of each camera–lens pair, including optical distortion, were calculated from 50 to 100 images of a flat, 1.2 × 0.7 m checkerboard that filled the field of view of each camera. To fill the view and achieve a sharp image, we increased sharpness over a larger depth of field by further closing the aperture; changing aperture has a negligible effect on the pin-hole calibration model. (ii) The extrinsic parameters of each camera set—position, orientation and scale—were calculated from images of a visually textured board with corner markers at known distances from each other to define scale. (iii) As the pattern on the board could only be seen from a single set of cameras at once, we aligned the upper and lower sets of cameras using a T-shaped wand with spherical reference points. Finally, a coordinate system was defined using an L-shaped wand whose axes pointed along and across the flight corridor, in the plane normal to gravity.

### 2.4. Estimating camera calibration accuracy

Reconstruction error has two sources: camera calibration and point matching. To estimate camera calibration error, we compared our reconstruction of a fibreglass bird model to a high-accuracy laser scan of the model (Romer Absolute Arm, RA-7525-SI, accuracy 0.063 mm). Model wingspan was 1.3 m, slightly greater than the goshawk's wingspan of approximately 1.1 m. The point cloud surfaces were generated with the model placed within the measurement volume at glide height. Point matching is dependent upon each object's visual features or visual ‘texture'. For the model, visual texture was added using a marker to make small dots of varying size, which enhanced effective point matching.

### 2.5. Wing surface measurement

Three-dimensional surface points of the birds were reconstructed using commercial photogrammetry software (Photoscan v. 1.3.5; Agisoft LLC, St Petersburg, Russia) and custom Python scripting. To accelerate processing, this was conducted using the Bluecrystal Phase 3 high-power computing facility at the University of Bristol. First, common image features were identified and matched between multiple views, providing an initial sparse reconstruction when combined with camera calibrations. The sparse reconstruction served as a foundation for disparity-map calculations between camera pairs. The detailed three-dimensional point cloud was then reconstructed from the disparity maps and camera calibrations. Each cloud point was assigned a greyscale value, based on the matched image pixels from which it was obtained.

### 2.6. Point cloud cleaning, tracking and segmenting

We wrote custom graphical user interfaces in order to remove spurious points from the point cloud and identify whether the point belonged to the wing, tail or ‘body' (head, torso and abdomen). We tracked the movement of each segment using an ‘iterative-closest-point' algorithm in MATLAB (The Mathworks, Inc., Natick, USA), which estimated the rigid-body transform, consisting of rotations and translations, that minimized the distance between two different point clouds of the same segment. For further details, see electronic supplementary material.

### 2.7. Sampling

We described 15, 14 and 13 glides for *T. alba*, *S. Aluco* and *A. gentilis,* respectively, once we removed glides with observable unsteadiness. As the birds glided steadily, we selected a single instance in time, in the centre of the measurement volume, to describe the properties of the glide. To compute time derivatives, we aligned the body and wings for a 50 ms window centred about the measurement instant and fitted a second-order polynomial to the data.

### 2.8. Alignment-transform decomposition

We deconstructed our alignment transforms into Euler angles and global translations. The order of rotations used to decompose the rotation matrices differed for the body and wings but were fundamentally similar. We calculated the rotations about the body in the order of yaw, pitch and then roll. Then, because long-axis rotation of the body—roll—is similar to long-axis rotation of the wing—pitch—we adjusted the rotation order for the wings to be sweep, elevation angle/dihedral and then pitch. This adjustment makes the axes order the same, if the long axes for the reference postures of the wing and body are parallel.

### 2.9. Coordinate systems

**Figure 2.**
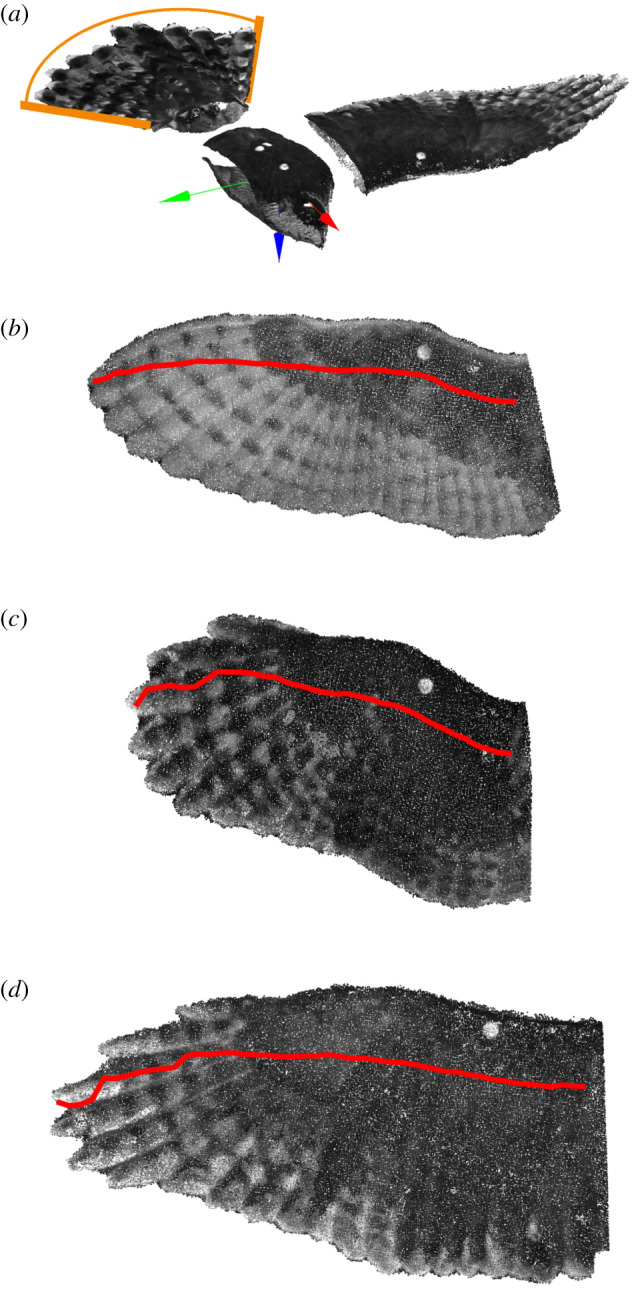
Reference postures for the body, wing and tail. (*a*) The segmented body, wing and tail. The plane of the tail is at 0° pitch and 0° roll; the spread angle of the tail is indicated by the arc (orange). The body and wing coordinate systems are aligned in rotation but offset in translation for visualization purposes. Translating along the axes indicates anterior (red), lateral (green) and ventral (blue) directions. Rotations about the anterior/red axis describe rolling the body or elevating wing angle; pitch is about the lateral/green axis and positive angles indicate trailing edge depression; and yaw or sweep are about the ventral/blue axis for the body and wing, respectively. For the tail, we only quantify pitch rotation. (*b*–*d*) The wings aligned to their respective coordinate systems for *T. alba* (*b*), *S. aluco* (*c*) and *A. gentilis* (*d*). The wing plane is parallel to the anterior and lateral axes (perpendicular to the ventral axis). We did not use morphology to define the long-axis of the wing and, therefore, its 0°-sweep state. Instead, the 0°-sweep state for the wing was defined as its average swept posture. In this way, when the wing is sectioned into chords, they are, on average, aligned with the uniform oncoming flow. The red line traces the quarter-chord positions along the span.

We created a coordinate system for the body to describe its gross movement. The axes of the body ran from: the base of the tail to the head, the body out to the right and the body out downward (figure 2*a*); rotations about each axis described roll, pitch up and yaw, respectively (further detailed in electronic supplementary material). We used the coordinate system to quantify the body's angle of attack using its pitch relative to the flow, the body's glide angle.

Our coordinate system for the wings described their gross movement and defined common spanwise and chordwise axes. For the wings, to keep movement descriptions consistent between the left and right wings, we mirrored the right wing about the sagittal plane of the body to align it with the left wing, which made rotations of the right wing use the same sign convention as that of the left wing, e.g. without mirroring the wings, positive wing sweep would describe the forward sweep of one wing and backward sweep of the other. Axes ran from the trailing edge to the leading edge—following the chord; distal to proximal; and dorsal to ventral—downward if the wing was horizontal (figure 2*a*); rotations about each axis describe wing elevation, pitch up and forward sweep. We defined the axis aligned with the wing chord based on the average flow orientation relative to the wings—and not based on wing morphology—this resulted in the average wing sweep equalling zero (figure 2; further detailed in electronic supplementary material, Methods). By aligning the wings and placing them in the wing-coordinate system, we could determine how chord properties, at the same position of the wing, changed with flight speed.

### 2.10. Computing wing parameters

To compute the chord profiles across the wing, we transformed the wings to their common wing-coordinate system, and then sectioned the wing at 1 cm intervals along the span. Wing chords were only computed if the whole chord belonged to the segmented wing; this removed the most-proximal sections at the wing root where portions of the chord would contain points belonging to the body. Having sectioned the wing, we smoothed the point cloud to identify the chord profile using the local three-dimensional geometry (see electronic supplementary material, methods).

After smoothing the chord profile, we processed the parameters of the wing chords by first computing the chord line, and then the mean line of the chord. We computed the chord line by identifying the leading edge as the point along the wing surface with greatest curvature occurring within the anterior half of the section. We then used a curvilinear-coordinate system to identify the mean line between the upper and lower surfaces, by using the proportional distance travelled along both surfaces from the leading edge to the trailing edge. From the chord line and mean line, we computed chord pitch, quarter-chord position, and the chordwise thickness and camber distribution.

## 3. Results

Throughout the results, we will quantify flight parameters and wing morphology metrics for *T. alba*, *S. aluco* and *A. gentilis.* To refrain from frequently repeating each species, when all three birds are described together, we will maintain this order, and only repeat the species names once for each section. We report mean ± s.e.m. unless stated otherwise, and we will repeat this once for each section.

### 3.1. Camera calibration accuracy

We estimated reconstruction error attributable to camera calibration by comparing a high-accuracy laser scan of a bird-shaped physical model (scan accuracy = 0.063 mm) to its point cloud reconstruction. The median absolute error was 0.6 mm; 72% of all points were within 1 mm from the laser scan, and 95% of all points were within 2.2 mm (figure 1*b*). The average signed error was 0.022 ± 0.002 mm (mean ± s.e.m.).

### 3.2. Average configuration, in brief

While our focus is on the changes in wing, body and tail morphing that occurred with changing flight speed, we begin by highlighting a few features of the average flight morphology for reference. The average was constructed from 15, 14 and 13 gliding flights for *T. alba*, *S. aluco* and *A. gentilis*, respectively. The details we highlight focus on parameters consistent across the three birds. A more detailed description of the average morphology and configuration can be found in the electronic supplementary material.

The configuration of the birds suggested that these flights were not near maximum speed, as the wings were protracted forward and the tails spread wide. The average flight speeds were 7.7 ± 0.1, 6.1 ± 0.1 and 7.3 ± 0.2 m s^−1^ (mean ± s.e.m.). Wing protraction, here, describes the forward translational movement of the quarter-chord points (figure 2*b–d*). At these speeds, the fitted line along the wingspan through these quarter-chord points was angled forward by 4.5 ± 0.2°, 7.9 ± 0.4° and 0.8 ± 0.2°, and the tail spread so its feathers covered an arc of 35.8 ± 2.7°, 51.2 ± 2.6° and 90.7 ± 5.9°; well above the minimum observed arcs of 20.7°, 30.8° and 38.9°.

**Figure 3.**
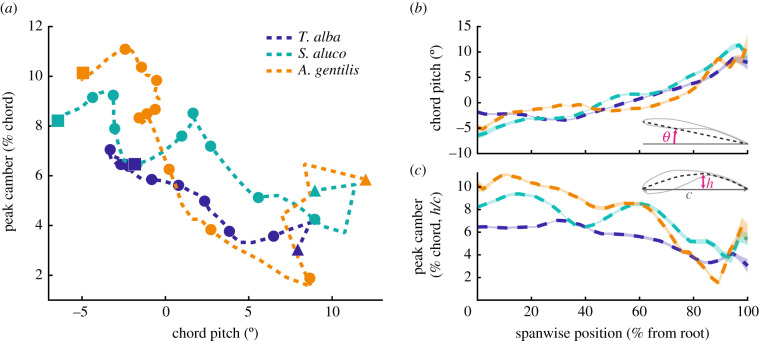
Mean chord pitch and peak camber are inversely related. (*a*) Near the wing root, wing camber is greatest and chord pitch is least (upper left quadrant). Wing camber remains high over a large portion of the wing, but as peak camber decreases, the pitch angle of each section increases. Eleven markers annotate 10% progressions along the wing length, from wing root (square on the left) to wingtip (triangle on the right). (*b*,*c*) Spanwise wing position plotted against chord pitch (*b*) and peak camber (*c*). Transparent colour patches encompass the standard error of the mean and are of similar thickness to the dashed mean line. Data reported for left and right wings combined.

The wings and tail operated at steep angles to the relative flow produced by the glide. The bodies' angles of attack relative to the wind (coordinate system in figure 2) were 2.9 ± 0.2°, 5.6 ± 0.6° and 6.1 ± 0.9°. The tail and wing were further inclined and pitched up relative to the body. The tail pitched relative to the body by 18.7 ± 0.6°, 27.9 ± 1.4° and 23.5 ± 1.9°. The plane of the wing pitched relative to the body by 1.3 ± 0.3°, 6.0 ± 0.5° and 8.4 ± 0.9°, and relative to that plane, wing twist resulted in the distal wing further pitching up by 9–11° (figure 3*b*).

Across the wingspan, the mean chord pitch and peak camber changed inversely (figure 3). Wing twist increased the chord angle of attack with distance from the shoulder, a configuration referred to as aerodynamic wash-in, and resulted in the distal wing operating at 10–15° higher angle of attack than proximal regions (figure 3*b*). However, the distal wing was less cambered than the proximal wing (figure 3*c*). The maximum wing camber across the entire wing was on average 7.0 ± 0.1, 9.4 ± 0.1 and 11.1 ± 0.1% of the chord length, and occurred proximal to the body at 33, 15 and 10% of wing length. Camber in the distal quarter of the wing was only 0.5–3.0% of the chord length.

**Figure 4.**
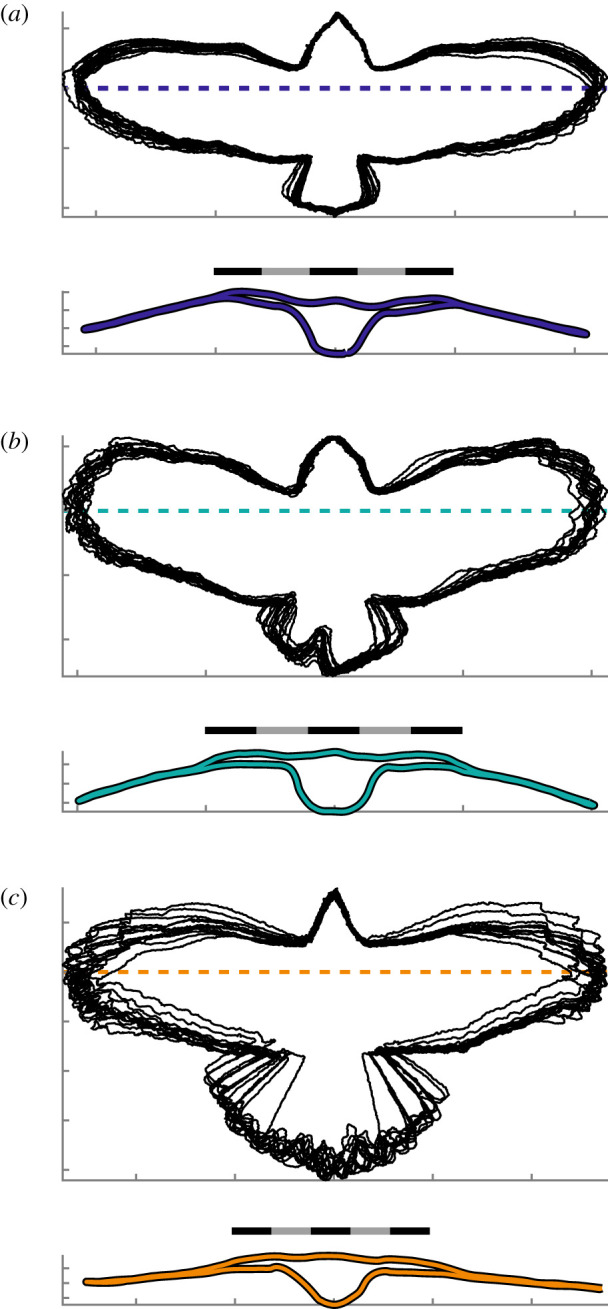
Ventral projections and a transverse slice through each bird. (*a*–*c*) (Upper panel) Projected outlines of birds aligned to their body coordinate system. (Lower panel) A transverse section through an individual glide posture with a representative degree of anhedral (within 1° of average). Location of transverse section denoted by the dashed line in the upper panel. (*a*) *T. alba* (*n* = 15 postures); (*b*) *S. aluco* (*n* = 14 postures); (*c*) *A. gentilis* (*n* = 13 postures). (*a*–*c*) Scale bars are 40 cm long.

**Figure 5.**
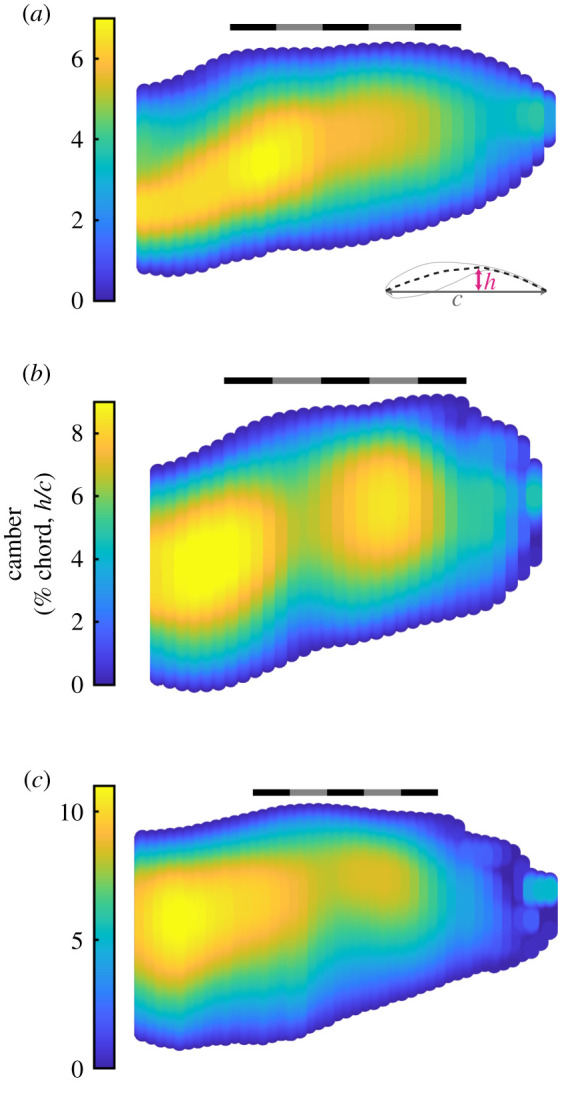
Mean camber distribution across the wings. (*a*–*c*) Point clouds of the average planform mapped with the average camber, quantified as a percentage of chord length. (*a*) *T. alba*; (*b*) *S. aluco*; (*c*) *A. gentilis*. (*a*–*c*) Scale bars are 20 cm long. Averages reported for left and right wings combined.

Other notable features were the interface between wing and body, wing anhedral, spanwise curvature and the absence of negative camber across the chords. The wing root intersected with the torso at the shoulder and formed a smooth dorsal surface (figure 4). The plane of the wings was depressed at an angle below the shoulder forming an anhedral configuration at angles of −7.1 ± 1.0°, −12.5 ± 0.6° and −6.5 ± 0.6° (values are negative to correspond with the rotation of the left wing). The wings did not protrude along a straight axis but were spanwise cambered with proximal concave-down curvature and distal concave-up curvature (figure 4; electronic supplementary material, figure S1B,C). Chordwise, where feathers overlap and form a smooth surface, the sections exhibited only positive camber (figure 5; see electronic supplementary material, figure S2 for uncertainty and alternative mapping).

### 3.3. Relationships with speed

We describe the wing and tail morphing over the flight speed range of 6.6–8.1, 5.2–6.9 and 6.3–8.6 m s^−1^, in *T. alba*, *S. aluco* and *A. gentilis*, respectively. For each, their fastest speed was 23, 33 and 36% greater than their slowest speed. The speed range of *T. alba* was substantially extended by a single slower flight, and with that flight excluded the fastest speed was only 10% greater than the slowest speed.

To determine changes with flight speed, we linearly regressed our measurements against speed. We report the slope of the linear relationship with flight speed as ‘units per m s^−1^’, and the mean ± s.e.m. of the slope. Some of the analysed metrics are expected to scale linearly with the lift coefficient, and, therefore, might scale better with inverse dynamic pressure, but over the observed ranges of flight speeds, the differences in quality of fit between speed and inverse dynamic pressure were small and regressing against inverse dynamic pressure did not affect our conclusions. We treated each bird independently in the regressions against speed, as their differing morphology should result in different scaling relationships with speed.

#### 3.3.1. Tail spread, wingspan and projected area

**Figure 6.**
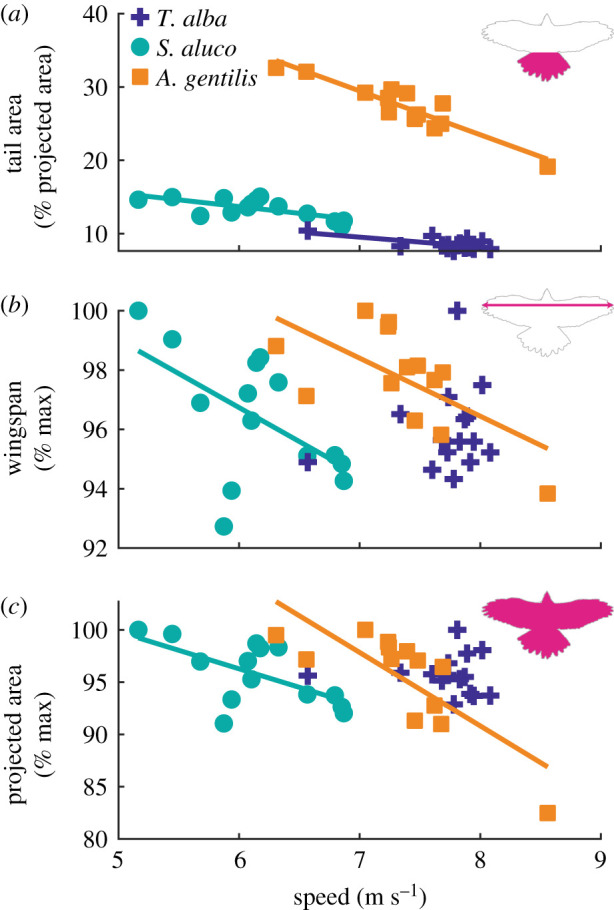
Relationships between tail area, wingspan and projected area with speed. (*a*) Percentage of the projected area represented by the tail decreased with speed. (*b*,*c*) Wingspan and whole-bird projected area decreased with increased speed in *S. aluco* and *A. gentilis*. Values were normalized by each bird's maximum observed value. (*a*–*c*) Lines indicate statistically significant relationships.

The tail contracted significantly with increased speed in all three birds. The arc angle covered by the tail feathers contracted by −28.1 ± 11.2°/(m s^−1^), −22.1 ± 5.9°/(m s^−1^) and −29.3 ± 4.7°/(m s^−1^) in *T. alba*, *S. aluco* and *A. gentilis*, respectively (mean ± s.e.m.; figure 2*a*; *p* = 0.03, 0.003, 7 × 10^−5^). While each of the three birds contracted their tail by relatively similar amounts with increased speed, the larger tail of *A. gentilis* resulted in a threefold greater proportional change in its ventrally projected area. The proportion of the area represented by the tail against that of the whole bird decreased by −1.3 ± 0.4%/(m s^−1^), −1.8 ± 0.5%/(m s^−1^) and −6.0 ± 0.7%/(m s^−1^) (figure 6*a*).

With increased speed, the wings also contracted in both *S. aluco* and *A. gentilis*. Relative to each bird's largest observed wingspan, wingspan decreased by −2.3 ± 1.0%/(m s^−1^) and −2.0 ± 0.7%/(m s^−1^), in *S. aluco* (*p* = 0.04) and *A. gentilis* (*p* = 0.02), respectively, and wingspan varied by 0.8 ± 1.1%/(m s^−1^) in *T. alba* (*p* = 0.48). With both wings and tail contracting, the ventrally projected area decreased with speed. Relative to each bird's largest observed area, area decreased by −3.5 ± 1.3%/(m s^−1^) and −7.0 ± 1.6%/(m s^−1^), in *S. aluco* (*p* = 0.02) and *A. gentilis* (*p* = 0.001), respectively, and area varied by −0.2 ± 1.4%/(m s^−1^) in *T. alba* (*p* = 0.91). The decrease in area was largely attributable to tail contraction: in *T. alba*, the tail was responsible for the overall decrease in projected area with increased speed, as planform area excluding the tail slightly increased; and in *S. aluco* and *A. gentilis*, the tail was responsible for 52% and 77% of the decrease in area with increased speed, respectively.

#### 3.3.2. Angle of attack

**Figure 7.**
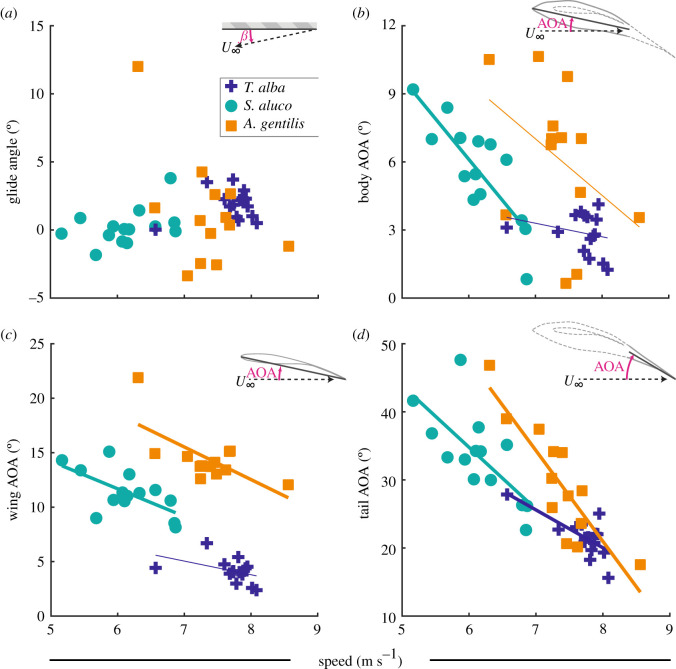
The relationships between speed and either glide angle or orientation relative to the flow (angle of attack: AOA) of the body, wing and tail. (*a*) The relationship between the glide angle and speed was not significant for any bird. (*b*–*d*) Angle of attack tended to decrease with increased speed. The angle of attack was defined by the pitch of the (*b*) body coordinate system; (*c*) wing coordinate system, i.e. the plane of the wing; and (*d*) plane of the tail. Thick lines indicate statistically significant relationships. (*b*,*c*) Thin lines are not statistically significant, but were used to compute residuals for other analyses.

When speed is varied, birds must compensate to maintain support for body weight. As area changes relatively little, either the glide angle must change dramatically or angle of attack must change inversely with speed in the whole bird, or its body, wings or tail. Change in the glide angle with increased speed was not statistically different from zero for any bird (figure 7*a*); the linear relationships with speed were: 0.5 ± 0.8°/(m s^−1^), 1.1 ± 0.7°/(m s^−1^) and −3.6 ± 1.9°/(m s^−1^) (mean ± s.e.m.; *p* = 0.56, 0.12, 0.08) for *T. alba*, *S. aluco* and *A. gentilis*, respectively.

The pitch of the tail changed with flight speed and not solely because of changes in the angle of attack of the body. If the whole bird's angle of attack changed, the angle-of-attack relationships with speed for the body, wings and tail would be equal. Instead, the tail's angle of attack decreased significantly more with speed than that of the body (figure 7*b,d*) in *T. alba* (*p* = 9 × 10^−6^), *S. aluco* (*p* = 0.001) and *A. gentilis* (*p* = 4 × 10^−6^). While the body's angle of attack decreased with speed significantly only in *S. aluco* by −3.6 ± 0.7°/(m s^−1^) (*p* = 3 × 10^−4^) and varied by −0.6 ± 0.7°/(m s^−1^) and −2.5 ± 1.6°/(m s^−1^) in *T. alba* (*p* = 0.38) and *A. gentilis*, respectively (*p* = 0.16), the tail's angle of attack decreased significantly with speed in all three birds, decreasing by −5.6 ± 1.5°/(m s^−1^), −9.1 ± 2.5°/(m s^−1^) and −13.3 ± 2.3° (m s^−1^) (*p* = 3 × 10^−3^, 4 × 10^−3^ and 1 × 10^−4^).

By contrast, the relationships between the plane of the wing's angle of attack and speed were not significantly different from the bodies' relationships in any bird (*p* = 0.36, 0.91 and 0.38). The wings' angles of attack decreased significantly with speed by −2.5 ± 0.8°/(m s^−1^) and −3.0 ± 1.0°/(m s^−1^), in *S. aluco* (*p* = 0.02) and *A. gentilis* (*p* = 0.01), respectively; and varied by −1.3 ± 0.7°/(m s^−1^) in *T. alba* (*p* = 0.11).

**Figure 8.**
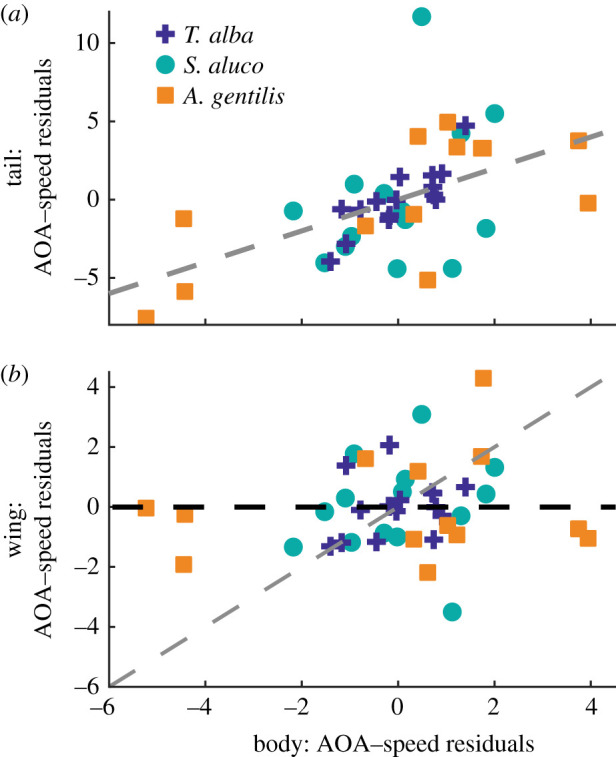
Relationships between residuals from the angle of attack–speed relationships. (*a*) The tail and body tended to deviate together, while (*b*) the wing did not deviate in response to body deviations. The wings compensated for the body's angle-of-attack deviations. Grey dashed lines indicate 1 : 1 relationships. Black dashed line indicates perfect pitch compensation at the shoulder.

If the wing and body can maintain a constant angle of attack relationship with speed, then shoulder pitch actuation is unnecessary for modulating angle of attack at different speeds; however, an important role for shoulder actuation may be to compensate for the body when it is pitched improperly for a given flight speed. Without compensation, deviations in the body's angle of attack from its predicted linear relationships with speed (i.e. the residuals) would lead to similar deviations for the wing and tail. Indeed, for the tail, when the residuals across all three birds were combined, tail-angle-of-attack residuals scaled by 1.10 ± 0.26° for every degree of body deviation, a relationship not significantly different from 1 to 1 (*p* = 0.70; figure 8*a*). This was not the case with the wing, which compensated for deviations from the body's expected angle of attack. Wing-angle-of-attack residuals scaled by 0.11 ± 0.12° for every degree of body deviation, significantly different from one to one scaling (*p* = 8 × 10^−9^; figure 8*b*), the shallow slope indicating substantial wing pitch compensation at the shoulder.

#### 3.3.3. Wing elevation angle and sweep

**Figure 9.**
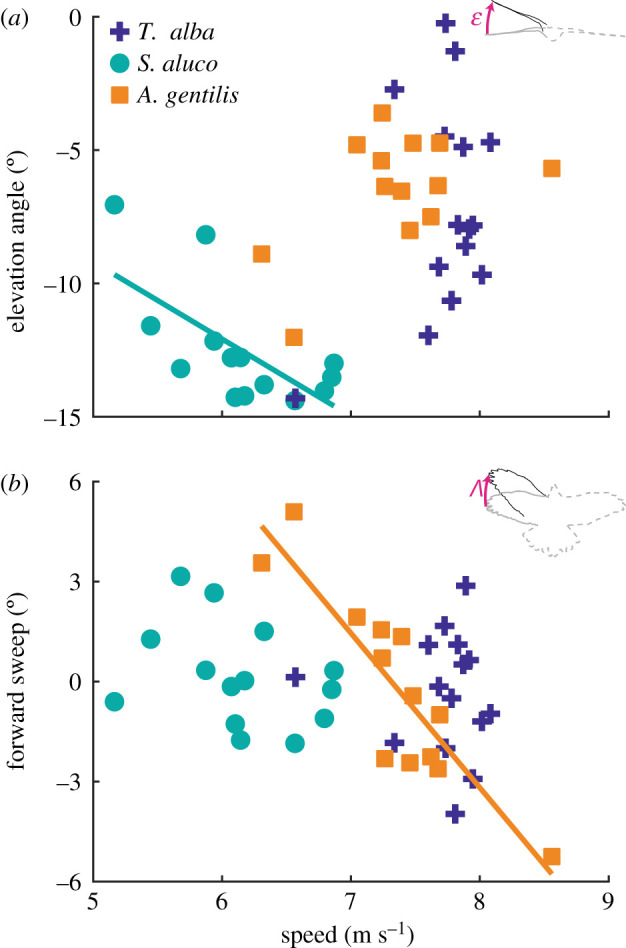
Changes in wing sweep and elevation angle with speed. (*a*) The wings depressed with increased speed in *S. aluco*. (*b*) The wings swept back with increased speed in *A. gentilis*. Lines indicate statistically significant relationships.

Rotations of the wing about the shoulder in sweep and elevation affect flight stability. The wings depressed with speed, increasing anhedral angle (reducing lateral stability), significantly only in *S. aluco* by −2.9 ± 0.9°/(m s^−1^) (mean ± s.e.m.; *p* = 0.009) and varied by 4.0 ± 2.8°/(m s^−1^) and ­­2.0 ± 1.1°/(m s^−1^) in *T. alba* (*p* = 0.18) and *A. gentilis* (*p* = 0.08), respectively (figure 9*a*). The wings swept backwards with speed significantly only in *A. gentilis* by −4.6 ± 0.7°/(m s^−1^) (*p* = 5 × 10^−5^) and varied by −0.3 ± 1.4°/(m s^−1^) and −1.0 ± 0.8°/(m s^−1^) in *T. alba* (*p* = 0.84) and *S. aluco* (*p* = 0.23), respectively (figure 9*b*). The consistency in wing-sweep posture of the two owls was also evident from the projections of the whole bird onto a ventral plane (figure 4).

#### 3.3.4. Wing planform

**Figure 10.**
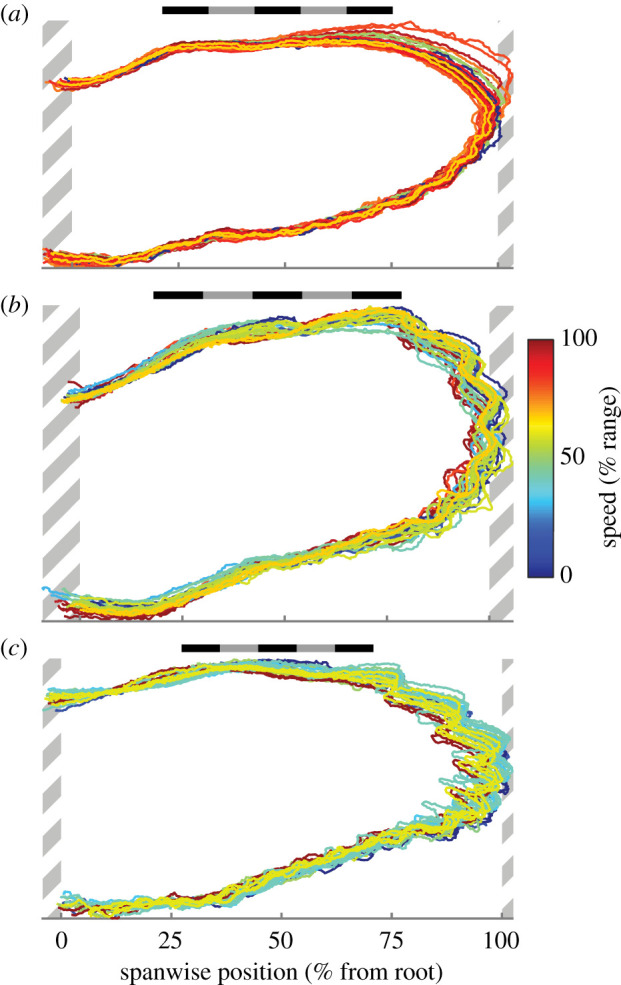
Planform similarity across speeds. The outlines of left and right wings after alignment to a common reference wing. Wing outlines are coloured according to flight speed as a percentage of the observed range. (*a*) *T. alba* (*n* = 30 wings); (*b*) *S. aluco* (*n* = 28 wings); (*c*) *A. gentilis* (*n* = 26 wings). Hatched areas are regions of wing not included in analysis and contain fewer than one-third of the wings. Scale bars are 20 cm long.

The wings maintained a similarly sized and shaped planform across all speeds, once aligned to a common coordinate system. The region of the planform with greatest change was the distal portion of the wing (figure 10). In *T. alba*, the planforms changed in a manner consistent with the second digit actuating and abducting the 10th primary feather anteriorly [13], extending the leading edge (figure 10*a*); extension of the distal leading edge in *T. alba* did not exhibit a relationship with speed (*p* = 0.82).

#### 3.3.5. Wing shape: spanwise distributions of twist/pitch

**Figure 11.**
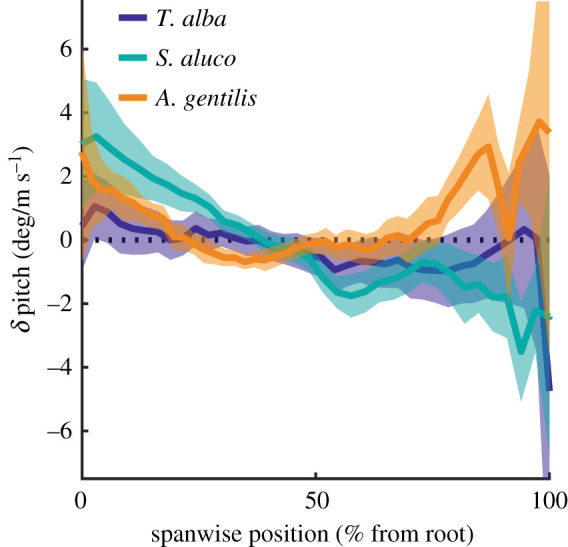
Wings twisted with speed. Chord pitch changed with flight speed across the wing length. Lines indicate means; colour patches indicate 95% confidence intervals; black dashed line indicates zero change in pitch with speed.

The wings of *S. aluco* and *A. gentilis* twisted significantly with increased speed (figure 11). The proximal wing pitched up with speed in both *S. aluco* and *A. gentilis*, while distally, the wing pitched down in *S. aluco* and up in *A. gentilis*. *T. alba* exhibited a similar pattern to *S. aluco*, but wing twist did not change significantly with speed along the wing and was generally of a lower magnitude.

#### 3.3.6. Wing shape: camber

**Figure 12.**
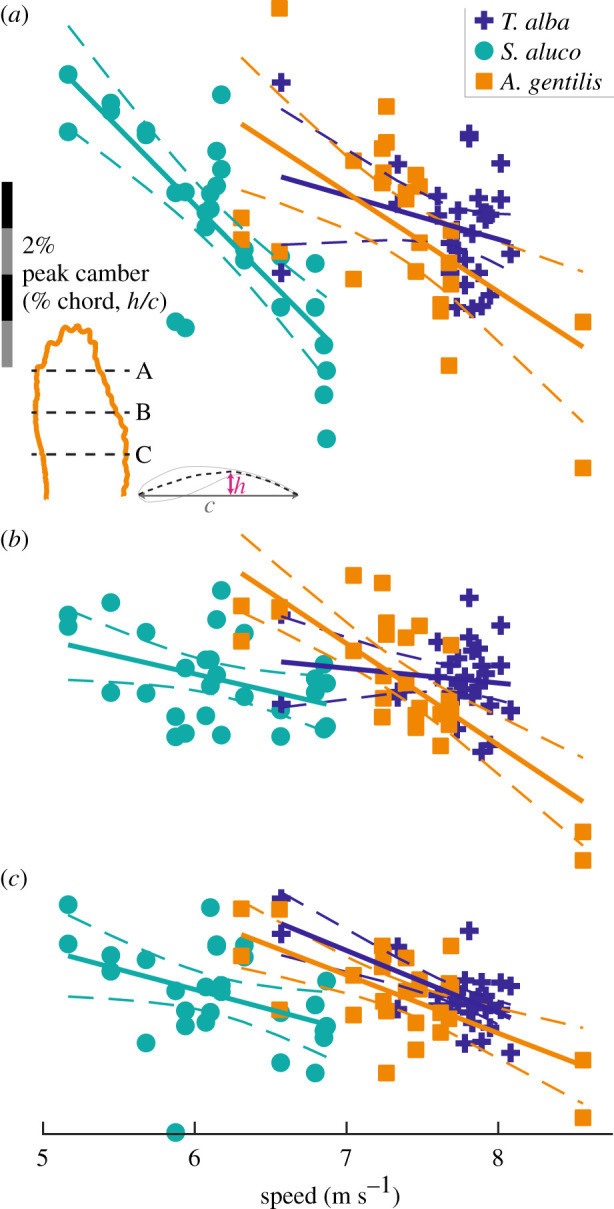
The relationship between peak camber and speed across three chords. The mean relationships plotted as solid lines, 95% confidence intervals plotted as dashed lines. Spanwise position of chords at (*a*) 75%, (*b*) 50% and (*c*) 25% wing length, indicated by black dashed lines in inset (*a*). Scale bar for peak camber in (*a*) applies to all panels. The left and right wings included separately as individual data points.

**Figure 13.**
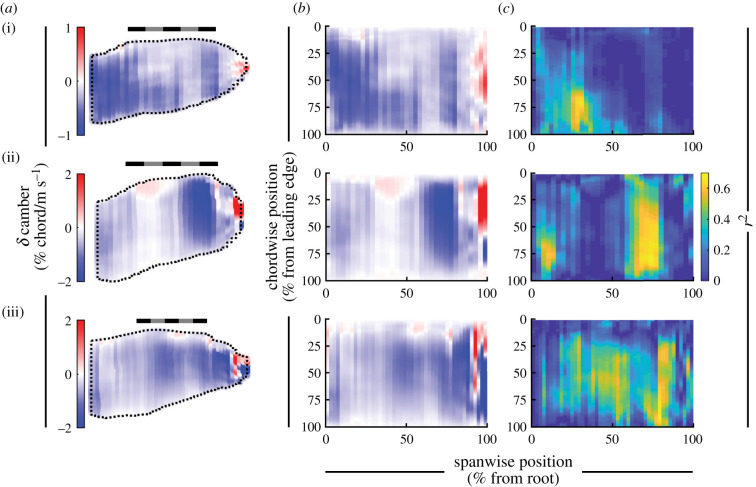
Wings reduced camber with speed. (*a*,*b*) The change in camber across the wing as a percentage of chord length, mapped to an average wing planform (*a*) or rectangle (*b*). (*c*) The distribution of the coefficient of determination (*r*^2^) for the relationship between camber and speed across the wing, mapped to a rectangle. (i) *T. alba*; (ii) *S. aluco*; (iii) *A. gentilis*. (*a*) Scale bars are 20 cm long.

Wing camber generally decreased with increased flight speed throughout the wing in all birds (figures 12 and 13). Figure 12 displays the relationship between peak camber and speed for three chords, and figure 13 plots the slope of the relationship between camber and speed throughout the wing. Camber decreased (i.e. flattened) with speed across 89, 85 and 89% of the planform area, and decreased by more than −0.5% chord/(m s^−1^) across 24%, 36% and 49% of the planform area in *T. alba*, *S. aluco* and *A. gentilis*, respectively. If we only examine wing regions with at least moderate coefficients of determination (*r*^2^ > 0.25), then camber decreased by more than −0.5% chord/(m s^−1^) across 64, 75 and 78% of the remaining planform. The small regions where wing camber increased were generally at the tips of the distal feathers, and, in *S. aluco*, near the wrist (figure 13).

#### 3.3.7. Wing shape: out-of-plane morphing

**Figure 14.**
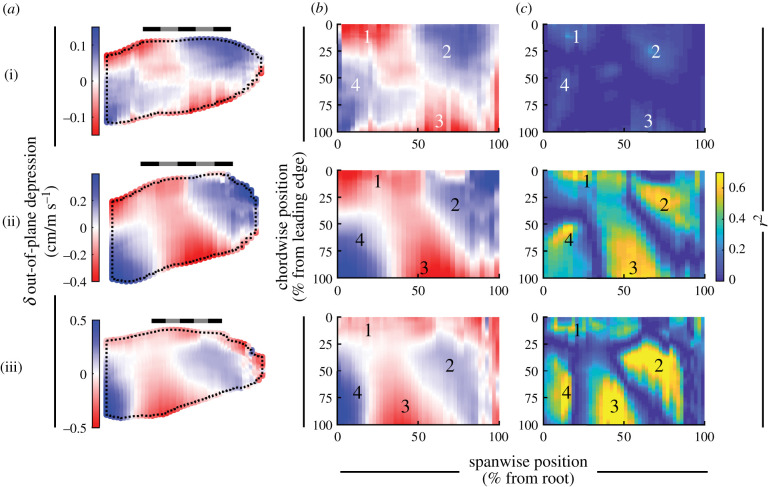
Wings deformed in a saddle pattern with speed. (*a*,*b*) The out-of-plane depression (rise: negative) of the wing mapped to an average wing planform (*a*) or rectangle (*b*). (*c*) The distribution of the coefficient of determination (*r*^2^) for the relationship between the out-of-plane movement and speed across the wing, mapped to a rectangle. (i) *T. alba*; (ii) *S. aluco*; (iii) *A. gentilis*. (*a*) Scale bars are 20 cm long. (*b*,*c*) Labels ‘1'–‘4' indicate regions of deformation consistent across all three birds. Colour map flipped to retain depressive movements as blue, consistent with figure 13.

All three birds exhibited similar patterns of out-of-plane wing movement with increased speed (figure 14). Out-of-plane morphing represented the cumulative effects of twist (figure 11), camber (figure 13) and spanwise changes in the curvature of the wing with speed (electronic supplementary material, figure S3*b*); we excluded changes in thickness (electronic supplementary material, figure S4). Among all three birds, we observed four consistent regions. Two regions depressed and two regions rose in height (figure 14*b*,*c*). These four regions form a saddle pattern across the wing, with one axis along the wing depressing (labels 2 and 4) and the perpendicular axis rising (labels 1 and 3). Each of these wing domains exhibited relatively high coefficients of determination when regressed against speed (*r*^2^); although coefficients of determination were generally low across the whole wing in *T. alba*.

### 3.4. Geometries and flight parameters

**Figure 15.**
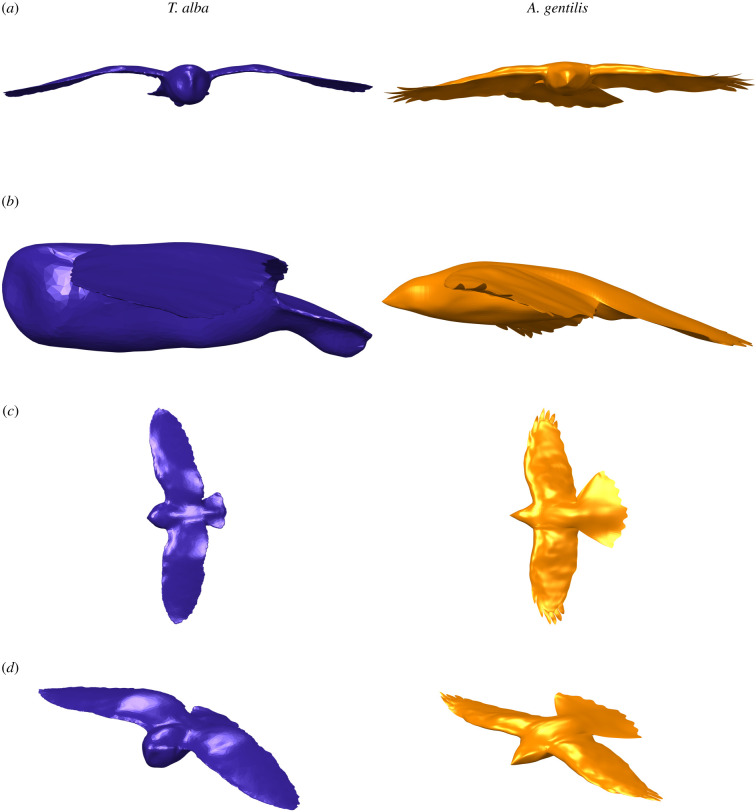
Three-dimensional geometries of *T. alba* (left) and *A. gentilis* (right). Views are (*a*) front, (*b*) left, (*c*) above and (*d*) isometric. Geometries available in electronic supplementary material.

In addition to the avian geometry parameters that we have measured, we have also included one reconstructed geometry each for *T. alba* and *A. gentilis* (figure 15) captured from the reconstruction of an individual flight. These are included within the electronic supplementary material to allow others to experiment and simulate with accurately reconstructed avian geometries. Flight speeds were 7.89 m s^−1^ and 7.46 m s^−1^, for *T. alba* and *A. gentilis*, respectively; 2 and 3% faster than the birds' average flight speeds (table 1).

**Table 1.** Glide velocity and acceleration for reconstructed geometries.

	anterior	lateral	ventral
*T. alba*: velocity (m s^−1^)	7.87	0.48	0.39
*T. alba*: acceleration (m s^−2^)	0.48	−0.1	−0.26
*A. gentilis*: velocity (m s^−1^)	7.45	0.24	0.08
*A. gentilis*: acceleration (m s^−2^)	0.19	0.47	−0.77

## 4. Discussion

We established the detailed in-flight wing morphology of three gliding raptors. We observed consistent patterns with speed in angle of attack, wing posture, wing and tail planform, and out-of-plane changes in the wing. With these details, we can better approach avian gliding flight integratively and comprehensively. Among our findings, we highlight how (i) the shoulder allows the wings to maintain a consistent angle of attack relationship with speed (figures 7 and 8), and allows the bird's body, which acts as an aerofoil, to pitch with minimal constraints; (ii) tail contraction and pitch (figures 6*a* and 7*d*) were consistent with drag minimization [10]; (iii) rigid-body movements at the shoulder account for the majority of changes in wing planform (figure 10), and how tail contraction accounts for as much area change as wing contraction; (iv) the tail might be responsible for changing wing camber through redistributing aerodynamic load; and (v) the geometries provided offer a tool for both simulation and experimentation to address these and many other questions aimed at understanding avian wing morphing.

### 4.1. The shoulder: compensates for body pitch deviation; not essential for speed change

The shoulder decouples wing pitch from body pitch, but pitch actuation may be relatively simple. Unlike the wing with its many linkages that impose and facilitate coordinated movement [15], the body is linked to the wing only at the shoulder and can operate relatively independently over the shoulder's substantial range of motion [20]. This degree of freedom could allow the wing and body to have different angle-of-attack relationships with speed, which theoretically could reduce drag [11], at the cost of additional control complexity. However, we could not reject the hypothesis, in any of the three birds, that, on average, the body and wing pitched together with speed. Therefore, the predominant role of wing pitch actuators at the shoulder may be to remain relatively rigid and adjust only when the body deviates from the expected relationship with speed, whether intentional or compensatory. Indeed, shoulder pitch maintained the wings' angle of attack relationship with speed with reduced variance (figures 7*c* and 8*b*). The shoulder, therefore, either compensated for the body's deviations in pitch, or allowed the aerofoil of the body [21] to modulate aerodynamic force and torque independent from the wing.

### 4.2. Change in tail size and pitch are consistent with drag minimization

With decreased speed, all three birds increased both the size of the tail (figure 6*a*) and its angle of attack (figure 7*d*), consistent with drag minimization [11]. If weight support is maintained, slower flight requires greater lift coefficients, obtainable through increasing angle of attack, and/or greater lifting area. No morphing is required if the whole bird pitches up to increase angle of attack; however, this leads to less efficient flight than morphing [22]. When morphing, it is only necessary for either pitch or area to change to support body weight, changing both is not required. However, previous simulation of our *T. alba* geometry with artificial manipulation of the tail demonstrated that slower flight required both greater tail pitch and tail spread to adopt the minimum drag configuration while providing body weight support, which is consistent with our observations of all three birds [10].

### 4.3. Morphing: in-plane shape and area change of the wing and tail

In-plane morphing of the wing was relatively subtle compared to that of the tail. The wing planforms varied among the flights (figure 4), but after accounting for movement at the shoulder, wing alignment demonstrated relatively consistent planforms (figure 10). The wings contracted with speed (figure 6*b*) as has been observed in other birds [3,4,23], but among our flights, in-plane movements did not lead to large changes in wing planform size or shape. By contrast, the tail contracted significantly with speed, and while it represented a modest proportion of the ventrally projected whole-bird area, changes in tail area accounted for a substantial proportion of the change in area. The size of the tail equated to 9%, 13%, and 27% of the projected area in *T. alba*, *S. aluco* and *A. gentilis*, but accounted for 24%, 39%, and 72% of the contraction in area.

### 4.4. Does camber reduce with speed as a result of tail morphing?

In studies of camber-morphing wings, increased camber is consistently beneficial at low speeds or high angles of attack, but, as speed increases and the lift coefficient requirement reduces, camber should decrease for efficient flight [9,24,25]. As changing camber with speed is beneficial, it is perhaps not surprising that it occurs in birds, but the mechanism by which camber changes is not clear, as there is relatively little muscle to control wing feather deflection actively. We hypothesize that camber changes as an aeroelastic response, primarily driven by tail morphing that is balanced by whole-bird changes in angle of attack (figure 7*b*).

Aeroelasticity can modulate the wing camber of flexible aerofoils through changes in the pressure difference between the upper and lower wing surfaces. Increased pressure differential increases the deflection of the trailing edge and reduces camber [26]. A change in the magnitude or distribution of the pressure difference around the relatively flexible wing of the bird could, therefore, change camber without direct muscular control.

**Figure 16.**
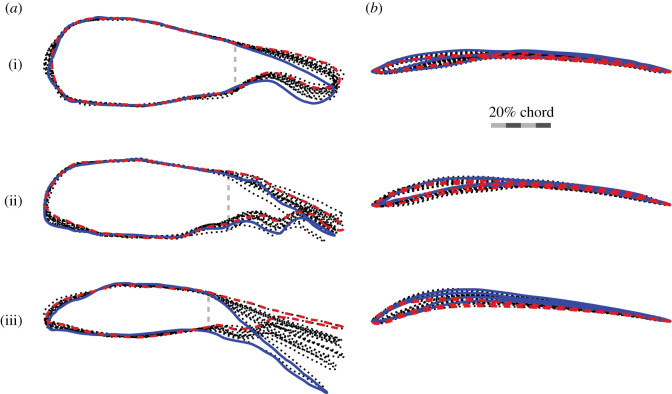
Chord profiles of the body change substantially with tail pitch. The chord profiles through (*a*) the body, tail and feet (sagittal section), and (*b*) midway along the wing (parasagittal section). Outlines are coloured for speed extremes (fastest: red dashed line; slowest: blue solid line). (i) *T. alba* (*n* = 15); (ii) *S. aluco* (*n* = 14); (iii) *A. gentilis* (*n* = 13). (*a*) Vertical grey dashed lines indicate body-to-tail transition. (*b*) Sample sizes are doubled as they show the left- and right-wing outlines. Wing sections are aligned to the chord line and scaled to chord length.

The effect of tail morphing is to predominantly change the effectiveness of the body as an aerofoil [10]. For example, in *T. alba*, whose tail is smallest proportionally, the differential pressure between the upper and lower tail surfaces accounts for only 3% of body weight, however, decreasing tail pitch by merely 5° decreases total aerodynamic force generation by 5%, more than what is supported solely by the tail [10]. In other words, the tail does not act as a wing; instead, it acts to modulate the body's aerofoil configuration (figure 16*a*).

Tail spread and pitch contribute substantially to both lifting area and lift distribution, and likely make tail action the dominant active morphing mechanism for modifying the distribution of pressure across the wing that results in changes in wing camber. With increased speed, decreases in tail area were proportionally greater than decreases in wing area (figure 6). Additionally, with increased speed, decreases in tail pitch substantially reduced the camber of the body-and-tail section (figure 16*a*), decreasing its lift production [25], suggesting that the lift produced by the wings must increase to maintain weight support—achieved through whole-bird changes in angle of attack, not through shoulder actuation. We estimate that the decrease in wing area did much less to increase aerodynamic pressure on the wings than either the reduction in tail spread or decrease in tail pitch. Computational results from transient fluid dynamics simulations of the included *T. alba* geometry suggest that the change in total lift with increased speed would have decreased by approximately 10% from tail action (spread: −28°/(m s^−1^); pitch: −5°/(m s^−1^)) [10], multiple factors greater than that of decreasing wing area (less than or equal to 3%/(m s^−1^) for each bird). This would suggest that tail action improves flight efficiency both by actively and directly modifying the geometry of the bird, and by passively and indirectly influencing wing camber through changing the pressure distribution acting on the wings (figure 16).

### 4.5. Future considerations

The geometries provided will help to address questions of raptor flight at and around the observed speeds. Morphing the geometries and examining their aerodynamic performance will elucidate whether the morphology supports efficiency, maximal loading, stability or may be a comprised design borne of evolutionary trade-offs with other requirements. Simulation of unloaded wings suggests optimization for maximal loading [27], but when wings are loaded, the aeroelastically deformed wings may support new conclusions. All three birds must achieve effective flapping flight too, and the aerodynamic optima differ between gliding and flapping flight [28]. We expect that the provision of these geometries will accelerate our capacity to address questions about avian gliding flight. We anticipate that aerodynamic results, whether through simulations [10] or aerodynamic measurements of three-dimensional printed models [19], will produce similar wakes as those observed in these same individuals [11] (note the *S. aluco* individuals differ between these studies).

## 5. Conclusion

We presented accurate and detailed reconstructions of the body, wing and tail morphology during gliding flight. The three birds shared common spanwise patterns of wing twist, an inverse relationship between twist and peak camber, and held their wings depressed below their shoulder in an anhedral configuration. Shoulder elevation and sweep controlled much of the planform, and after aligning the wing to a common coordinate system, the wing's in-plane configuration was remarkably similar. Shoulder pitch was critical to maintaining control over the wing's angle of attack.

Tail morphing supports minimizing drag, both by modulating aerodynamic performance and, we postulate, through actively manipulating the aeroelastic behaviour of the wing. The tail pitched up and contracted with increased speed, matching the predicted behaviour from computational studies. Further, the same tail movements also likely increased loading on the wings, changing wing camber through aeroelastic deflection. The birds reduced camber with increased speed, which is the expected pattern based on studies of efficient flight with variable-camber wings. This would suggest that through solely tail control, birds control multiple portions of their morphology through coupling and reduce the need for actuators in their wing.

We have described an array of aerodynamic traits that as a whole form a complex and dynamic solution for flight. Further, unlike traditional fixed-wing aircraft, these traits change with speed, all of which will require substantial analytical studies. Our provided geometries of *T. alba* and *A. gentilis* should provide a strong foundation and powerful initial tool to accelerate our understanding of avian wing morphology and morphing, as a reference for bioinspired engineering.

## Data accessibility

The data are provided in electronic supplementary material [29].
